# Qualitative aspects of developmental language impairment relate to language and literacy outcome in adulthood

**DOI:** 10.1080/13682820802708080

**Published:** 2009-07

**Authors:** Andrew J O Whitehouse, E A Line, Helen J Watt, Dorothy V M Bishop

**Affiliations:** Department of Experimental Psychology, University of OxfordOxford, UK

**Keywords:** specific language impairment, pragmatic language impairment, autism, language, literacy, outcome

## Abstract

*Background:* Developmental language disorder is a heterogeneous diagnostic category. Little research has compared the long-term outcomes of children with different subtypes of language impairment.

*Aims:* To determine whether the pattern of language impairment in childhood related to language and literacy outcomes in adulthood.

*Methods & Procedures:* Adults who took part in previous studies as children were traced. There were four groups of participants, each with a different childhood diagnosis: specific language impairment (SLI; *n* = 19, mean age at follow-up = 24;8), pragmatic language impairment (PLI; *n* = 7, mean age at follow-up = 22;3), autism spectrum disorder (ASD; *n* = 11; mean age at follow-up = 21;9), and no childhood diagnosis (typical; *n* = 12; mean age at follow-up = 21;6). Participants were administered a battery of language and literacy tests.

*Outcomes & Results:* Adults with a history of SLI had persisting language impairment as well as considerable literacy difficulties. Pragmatic deficits also appeared to develop over time in these individuals. The PLI group had enduring difficulties with language use, but presented with relatively intact language and literacy skills. Although there were some similarities in the language profile of the PLI and ASD groups, the ASD group was found to have more severe pragmatic deficits and parent-reported linguistic difficulties in conversational speech.

*Conclusions & Implications:* The pattern of deficits observed in different subtypes of developmental language disorder persists into adulthood. The findings highlight the importance of a wide-ranging clinical assessment in childhood, which may provide an indication of outcome in adulthood.

**What this paper adds**

**What is already known on this subject**

Specific language impairment (SLI) is a heterogeneous diagnostic category, incorporating a range of expressive and/or receptive language problems. A number of studies have found that the language and literacy difficulties of children with SLI often persist into adulthood. However, little is known about the outcome of individuals with different subtypes of language impairment, such as those with pragmatic language impairment (PLI).

**What this study adds**

This study is the first to compare the language profiles in adulthood of children diagnosed with either SLI or PLI. Childhood language profile persisted over time; the majority of children with SLI showed lasting deficits in structural language and literacy ability, while the children with PLI were found to have enduring pragmatic difficulties. Intriguingly, social language difficulties appeared to develop over time in the children with SLI. These findings not only highlight the distinction between SLI and PLI, but also the importance of a wide-ranging clinical assessment in childhood in order to identify areas likely to be in need of current or future intervention.

## Introduction

It is estimated that as many as 7% of children exhibit some form of unexplained language impairment (Tomblin *et al.*^1997^), yet little is known about the progression of developmental language disorders across the life span. Although many preschool language problems resolve, when language impairment persists to school age the prognosis is not as good. The few longer-term investigations have found that language impairments often remain in adolescence (Clark *et al.*^2007^, Conti-Ramsden and Durkin 2007, Stothard *et al.*^1998^) and adulthood (Johnson *et al.*^1999^, Clegg *et al.*^2005^), often with concomitant literacy deficits (Clegg *et al.*^2005^, Snowling *et al.*^2000^). However, a consistent finding among these studies has been the variability of outcome.

Perhaps the two best-documented cohorts are those followed by Beitchman in Canada and Rutter in the UK. The Beitchman sample, identified at age 5 years through a screening of English-speaking kindergartens (Beitchman *et al.* 1989), was tracked through adolescence (Beitchman *et al.* 1996) to early adulthood (Young *et al.* 2002). At the latest follow-up (*n* = 49; mean age = 19 years; standard deviation (SD) = 0.44 years), many individuals continued to show persisting language difficulties as well as considerable literacy difficulties (Young *et al.* 2002). However, around 30% of the sample showed no difficulties with the psychometric language tests (Johnson *et al.*^1999^) and 70% had reading ability within the normal range (Young *et al.* 2002). The Rutter sample was recruited in early childhood, along with a group of children with autism, from paediatric units attached to hospitals (Bartak *et al.*^1975^). When these individuals were followed-up in early adulthood (Mawhood *et al.* 2000) and later, in their mid-30s (Clegg *et al.*^2005^), the majority showed persisting linguistic deficits. However, once again, there were a minority of participants who had language abilities that bordered or were within the normal range (Clegg *et al.*^2005^). An additional finding at follow-up was that the language impairments of many of the SLI participants was similar to that observed in the autism participants, with deficits in language use as well as language structure (Mawhood *et al.* 2000).

SLI is a heterogeneous category, encompassing a variety of receptive and/or expressive speech/language difficulties. It is possible that the variability in outcome relates to differences in childhood speech/language profiles. There is some existing evidence for this proposal. For example, children with speech disorders are known to present with considerably better language and literacy ability in adulthood than those with a primary impairment in language ability (Johnson *et al.*^1999^, King *et al.*^1982^). However, there is a dearth of research that has compared the outcome of children with different subtypes of language impairment.

One taxonomy proposed by Rapin and Allen (1983) and Bishop and Rosenbloom (1987) distinguished between children with linguistic problems (structural deficits), and those whose difficulties are predominantly in using language appropriately (pragmatic deficits).^1^ Children in the former group correspond to traditional notions of specific language impairment (SLI), with difficulty in using or understanding morphology and/or syntax. Children in the latter category have superficially normal language development, but are verbose, display difficulty in using pragmatic cues in conversation and have problems producing and comprehending connected discourse. Originally referred to as Semantic–Pragmatic Disorder, there has been a recent shift towards the alternative diagnostic label of Pragmatic Language Impairment (PLI) (Bishop and Norbury 2002). This shift has come about due to evidence that pragmatic difficulties do not always occur with semantic impairments (Bishop 1998). Although the current paper will adopt PLI in reference to this syndrome, it is important to note that this term is not currently recognized in international diagnostic guidelines.

Although there is evidence supporting the differentiation of PLI and SLI in childhood (Bishop and Norbury 2002, Botting and Conti-Ramsden 2003), most longitudinal studies have not drawn this distinction. It therefore remains unclear how stable these subtypes of developmental language disorder are across the life span. This question has been complicated by the question of whether PLI should be regarded as a subtype of developmental language disorder, or would be better categorized with the pervasive developmental disorders (Boucher 1998). If the latter is the case, then we might expect to see outcomes of children with PLI more closely resembling those of children with autism than those of children with SLI. Information about language outcomes can help throw further light on this question.

The primary aim of this study was to determine whether the qualitative profile of language impairment in childhood — that is, whether impairments are predominantly in the structural (SLI) or pragmatic language domain (PLI) — is indicative of language and literacy outcome in adulthood. Our working hypothesis was that the pattern of deficits would persist into adulthood, so that those who had typical SLI in childhood would continue to have limitations on conventional tests of language processing and verbal ability, whereas those with a history of PLI would do much better on formal tests, but continue to show signs of inappropriate language usage. Given the high level of reading difficulties reported among children with typical SLI (Catts *et al.*^2005^), we further predicted that literacy problems would be more commonly seen in this group.

In order to obtain a large enough sample to address these questions, we combined data from participants who had originally taken part in one of six research projects conducted by Bishop and colleagues, and published in the period 1989–2004 (see Appendix A contains more information on these studies). Although the language measures obtained in childhood varied from study to study, all these participants had been classified as cases of either SLI or PLI when first seen.

At follow-up, the parents of these individuals were interviewed with the Autism Diagnostic Interview — Revised (Lord *et al.*^1994^), which asks for details about autistic symptomatology during early development. A proportion of these participants retrospectively met diagnostic criteria for autism in childhood — a finding we have argued reflects the broadening of autism diagnostic boundaries over the past two decades (Bishop *et al.*^2008^).^2^ We included these participants as a separate group in our study, because they could provide key information on the question of whether outcomes for children with PLI were more similar to those with autism or those with SLI.

In sum, the current paper reports the cognitive, language and literacy outcomes in adulthood of individuals in four groups: childhood diagnosis of SLI, childhood diagnosis of PLI, retrospective diagnosis of childhood autism and no childhood diagnosis. The psychosocial outcomes of these groups are presented in a companion paper (Whitehouse *et al.*submitted).

## Method

### Recruitment at initial testing (T1)

Clinical participants from each of the six cohorts (A to F) were initially recruited from schools around the UK that specialize in the education of children with communication disorders. A differential diagnosis of SLI or PLI had been made based upon teacher report and performance on standardized language tests (see Appendix A). Participants were deemed to have SLI if they scored within the normal range on a test of non-verbal ability, but at least 1 SD below the mean on one or more standardized language assessments. Although these are relatively liberal criteria for SLI, all children had undergone extensive diagnostic testing as condition of entry to their school. Pragmatic language ability was gauged through either teacher/therapist report or questionnaire assessment; these were the only methods available for determining pragmatic impairment in the 1980s and 1990s (Bishop and Rosenbloom 1987, Rapin and Allen 1983). Participants were considered to have PLI if they displayed a disproportionate amount of pragmatic language difficulties relative to structural language difficulties. Each cohort also included a group of typically developing children who were recruited from mainstream schools at T1 to act as control participants. These children had no history of developmental disorder or neurological impairment and all had English as their first language.

### Recruitment at follow-up testing (T2)

Clinical and control participants from cohorts A, B and C were located by the Office of National Statistics (UK), who traced participants from information on full name and date of birth. This procedure was approved by the Central University Research Ethics Committee of Oxford University. Of the 130 participants for whom these data were available, 51 language impaired participants (cohort A = 31, cohort B = 5, cohort C = 15) and 22 control participants (cohort B = 4, cohort C = 18) were successfully located and agreed to be sent information on the current study. Participants in the remaining three cohorts were contacted from our existing records (language impaired: cohort D = 16, cohort E = 15, cohort F = 12; controls: cohort D = 8). In total, 50 participants returned consent forms agreeing to participate in the current study. One control participant was excluded due to the presence of a significant head injury resulting from a motor vehicle accident, leaving a total of 49 participants who were seen at T2: 21 adults with an original diagnosis of SLI, 16 adults with an original diagnosis of PLI and 12 adults who were control participants at T1.

### Participants

According to the ADI-R interview conducted at T2, two participants in the SLI group and nine in the PLI group retrospectively met full criteria for autism in childhood (Bishop *et al.*^2008^,^3^). These participants were pooled to form a separate autism spectrum disorder (ASD) group. One further participant in the SLI group was unable to complete the psychometric battery due to the onset of psychiatric illness and was excluded from analysis. This left 18 participants in the SLI group, seven participants in the PLI group, eleven participants in the ASD group and twelve participants in the typical group. Participant details are presented in Table 1. There was no significant difference between the mean age of the four participant groups at T1, *F*(3, 44) = 1.56, *p* = 0.21, or T2, *F*(3, 44) = 1.97, *p* = 0.13.

**Table 1:** Mean (standard deviation) age and age range of participants at initial testing (T1) and at follow-up (T2). Also included is information about which study the participants took part in at T1

	Specific language impairment (SLI) (*n* = 18)	Pragmatic language impairment (PLI) (*n* = 7)	Autism spectrum disorder (ASD) (*n* = 11)	Typical (*n* = 12)
Age at T1	11;11 (3;0)	9;7 (2;3)	10;2 (3;2)	11;2 (2;10)
Range	6;1–15;7	7;0–13;9	6;2–15;11	7;0–15;7
Age at T2	24;8 (4;4)	22;3 (5;4)	21;9 (4;0)	21;6 (3;2)
Range	16;5–31;0	16;2–28;9	16;1–28;9	18;0–28;9
*Study cohort*				
A	8 M	2 M	1 M	–
B	–	1 M	1 M	–
C	1 M	1 F	4 M	1 M, 3 F
D	2 M, 5 F	–	1 M	3 M, 5 F
E	1 M	2 M	2 M	–
F	1 M	1 M	2 M	–

Note: M, male; F, female.

To compare characteristics of those who did and did not take part in this study, measures of non-verbal IQ and receptive language were converted to scaled scores with a mean of 100 and SD of 15 (for the exact measures used here, see Appendix A). Although it is not ideal to collapse data from different tests with different content, this allowed us to get a general idea of level of impairment. Those clinical participants who agreed to take part in the current study did not differ from the remainder of the clinical sample (including those we could not or did not contact) in terms of either non-verbal ability when first seen (mean = 100.42, SD = 11.92 for 120 non-participants and mean = 101.65, SD = 14.41 for 36 participants; *t*(154) = 0.52, *p* = 0.61) or receptive language level (mean = 80.1, SD = 13.52 for non-participants and mean = 78.1, SD = 15.91 for participants; *t*(154) = 0.74, *p* = 0.46). Similarly, the typically developing comparison participants in the current study did not differ from the remainder of the control sample at T1 on non-verbal ability (mean = 104.48, SD = 12.22 for 104 non-participants and mean = 102.17, SD = 8.91 for 12 participants; *t*(114) = 0.63, *p* = 0.53) and receptive language (mean = 105.07, SD = 10.51 for non-participants and mean = 110.17, SD = 11.51 for participants; *t*(114) = 1.58, *p* = 0.12).

A final analysis compared the childhood non-verbal ability and receptive language level of the four T2 participant groups. A one-way analysis of variance (ANOVA) found significant group difference in receptive language level, *F*(3, 44) = 10.22, *p*<0.01. The receptive language level of the typically developing group (mean = 109.42, SD = 12.15) was significantly greater than the SLI (*p*<0.01; mean = 78.11, SD = 16.67), ASD (*p*<0.01; mean = 78.27, SD = 22.59) and PLI groups (*p*<0.05; mean = 87.5, SD = 8.5). In comparison, there was no group difference in childhood non-verbal ability, *F*(3, 44) = 0.7, *p* = 0.56 (SLI: mean = 101.28, SD = 10.9; PLI: mean = 108.86, SD = 13.66; ASD: mean = 101.23, SD = 17.18, Typical: mean = 103.42, SD = 8.71).

### Participant ability at T1

Each cohort was administered assessments of non-verbal IQ and linguistic ability at T1 (see Appendix A). Non-verbal IQ was scaled to a mean of 100 and SD of 15. Participants' linguistic ability at T1 was measured by at least one expressive and one receptive measure. However, there was considerable variability in the exact psychometric tests administered from study to study. In order to provide a standardized measure of linguistic performance across the six cohorts at T1, participant performance on the linguistic tests at T1 were translated into a severity score. Each participant's performance was rated as demonstrating either good, fair or poor functioning (see Appendix B).

### Participant ability at T2

Participant ability at T2 was assessed with a wide range of psychometric tests in a session lasting roughly two hours. The session was conducted by one of the four authors (predominantly E. L. and A. W.), all of whom have extensive experience in administering psychometric assessments for research purposes. The chosen assessments covered both structural (articulation, expressive vocabulary, receptive grammar as well as narrative telling and understanding) and pragmatic language abilities. We also examined verbal short-term memory as well as phonological awareness. These abilities are known to be important for efficient language and literacy learning (Alloway *et al.*^2005^) and are typically impaired in children with SLI (Archibald and Gathercole 2006). Because language tests norms often do not extend to adults, raw scores were analysed in some cases.

#### Non-verbal IQ (NVIQ)

NVIQ was estimated with the block design and matrix reasoning subtests of the Wechsler Abbreviated Scale of Intelligence (WASI) (Wechsler and Chen 1999).

#### Structural language

Receptive language ability was assessed with the Test for Reception of Grammar — Electronic (TROG-E) (Bishop 2005) and vocabulary with the British Vocabulary Picture Scale — Second Edition (BPVS-II) (Dunn *et al.*^1997^). Raw scores on the TROG-E were converted to standard scores. The chronological age of the participants lay outside the range of standard scores for the BPVS-II and raw scores were analysed instead. Articulation ability was gauged with the Goldman–Fristoe Test of Articulation (Goldman and Fristoe 1986), for which the index was the raw error total. Participant responses on this test were audiotaped and scored offline by the researcher who administered the assessments. Where there was uncertainty as to a participant's response, the recording was heard by, and discussed with, another researcher until consensus on the response was achieved.

Participants also completed the beach story subtest of the Expression, Reception and Recall of Narrative Instrument (ERRNI) (Bishop 2004). This task required participants to familiarize themselves with a series of 15 pictures that told a narrative. Participants were then required to tell a story based on the pictures. After a 15–30-minute interval, participants retold the story, this time without the aid of the pictures. Following the retelling of the narrative, participants were asked a series of standardized questions relating to the story. This assessment measures: (1) the content of the initial narrative (initial telling); (2) the content of the delayed narrative (delayed telling); (3) the accuracy of answers to questions that assess literal and inferential understanding (comprehension); and (4) mean number of words in each utterance, averaged over the two narrative tellings (MLUw). Standard scores were computed for each measure. Narratives were transcribed by one of two researchers (E. L. and A. W.). To ensure accuracy in scoring, a random sample of 10% of the narrative tellings (*n* = 10) were transcribed by both E. L. and A. W. There was a high level of agreement in the content of the story transcriptions (91% of words were transcribed identically).

#### Phonological awareness

Phonological awareness was assessed with the spoonerisms subtest from the York Adult Assessment (Hatcher *et al.*^2002^). Participants were read aloud a name and their task was to create two new words by transposing the initial sounds of the first and last name, for example, Michael Jackson → Jichael Mackson. Because the name stimuli were well known to people in the UK, memory demands in this task were reduced. There were twelve names in total and participants were given one point for every first or last name they transposed correctly (that is, maximum of 24).

#### Verbal short term-memory (STM)

Verbal STM was measured with the repetition of nonsense words and memory for sentences subtests from the NEPSY (Korkman *et al.*^1998^). Scores are based on the number of syllables or words correctly recalled. Participant responses were audiotaped and scored offline. Once again, where a response was unclear, a second researcher was consulted until consensus was achieved. Since normative data on the NEPSY has an upper limit of 12, raw scores were analysed with higher scores indicating better performance (maximum for repetition of nonsense words = 46; maximum for memory for sentences = 34).

#### Pragmatic language

Pragmatic ability was assessed with the Communication Checklist — Adult (CC-A) (Whitehouse and Bishop 2009), a modified version of the *Children's Communication Checklist — Second Edition* (CCC-2) (Bishop 2003). The CC-A is completed by an adult who has regular contact (3–4 days per week) with the individual in question (for example, a friend, parent, etc.). CC-A maintains the same format of the CCC-2: there are 70 items (behavioural statements), which are divided into four subscales measuring structural language skills (that is, speech, syntax, semantics, coherence), four measuring pragmatic language skills (inappropriate initiation, stereotyped language, use of context, non-verbal communication) and two measuring other autistic-like behaviour (social relations and interests), each with seven items. Items have been modified in the CC-A so that they are suitable for adults.

Pilot testing of over 200 people aged 17–80 years has found that unimpaired adults very rarely are coded as other than optimal. Because of this, raw scores were used in the current study, with higher scores indicating greater impairment. A structural language composite (SLC) and pragmatic language composite (PLC) were computed by summing all items relating to the four structural language subscales and the four pragmatic language subscales respectively. ^4^On each of these scales, a higher score indicates greater levels of impairment. A structural–pragmatic mismatch score was calculated for each participant by subtracting the PLC from the SLC. A positive score is indicative of an individual with predominantly structural language difficulties, while a negative score would denote an individual with pragmatic/social difficulties that are disproportionate to structural language impairments.

#### Literacy

Spelling ability was assessed with the OSCCI spelling test, which is a simple dictation task devised by our research group (Whitehouse *et al.*^2007^). Participants are read a list consisting of forty words with either regular or irregular spelling. Participants are required to transcribe as many dictated words as possible in two minutes. One point was given for every correctly spelt item (a maximum of 40). Reading skills were assessed with the two subtests of the Test of Word Reading Efficiency (TOWRE) (Torgesen *et al.*^1999^): sight word efficiency, which assesses real word reading, and phonemic decoding efficiency, assessing non-word reading. Raw scores were converted to scaled scores based on American norms for adult readers.

Reading ability was further assessed with two subtests from the York Adult Assessment (Hatcher *et al.*^2002^). The nonsense passage reading test was a timed task in which participants were required to read a short passage consisting of nonsense words embedded amongst real words. For each of two passages, participants were scored on time and accuracy of pronunciation. In the proof reading task, participants were given a passage containing errors of punctuation, spelling and grammar. Participants were required to identify as many errors as they could in the shortest time possible. Scores were based on the number of errors identified (a maximum of 13) and the time taken to identify these errors.

### Structural language severity rating at T2

Outcome data at T2 were categorized into three categories to parallel the classification at T1. Participant's performance on two expressive (MLUw and the memory for sentences task) and two receptive language tasks (BPVS-II, TROG-E) at T2 were translated into a severity score based upon whether performance indicated a good, fair or poor level of functioning (see Appendix B).

## Results

### Cognitive and language functioning at T2

Standard scores on the WASI, TROG-E, the TOWRE subtests and the measures from the ERRNI were based around a mean of 100 and an SD of 15. Because of the considerable range in chronological age, each analysis was conducted with and without this variable as a covariate. The same patterns of significant differences were found (Table 2 presents group scores).

**Table 2:** Mean (standard deviation) performance of the four groups on the battery of cognitive and language tests at follow-up. Only statistically significant differences are presented

Domain	Assessment	Specific language impairment (SLI) (*n* = 18)	Pragmatic language impairment (PLI) (*n* = 7)	Autism spectrum disorder (ASD) (*n* = 11)	Typical (T) (*n* = 12)	Degrees of freedom (d.f.)	*F*	*p*	Partial eta^2^	Statistical significant differences
Non-verbal IQ	WASI (ss)	97 (16.95)	105.71 (16.58)	103.36 (19.35)	118.5 (8.61)		4.45	0.008	0.23	SLI<T
Structural language	Goldman–Fristoe (*n* errors)	4.06 (3.11)	1.43 (1.81)	1.64 (2.16)	0.67 (1.44)		5.64	0.001	0.28	SLI<T
	BPVS-II (rs)	130.06 (13.68)	127.43 (14.79)	122.73 (21)	146.08 (6.67)		5.53	0.003	0.27	(ASD = SLI)<T
	TROG-E (ss)	82.77 (13.57)	90.43 (24.33)	95.54 (11.56)	102 (4.35)		5.17	0.003	0.26	SLI<T
Narrative	Initial telling (ss)	88.00 (25.53)	99.14 (25.56)	89.18 (18.95)	104.5 (16.48)		1.64	0.19	0.1	No differences
	Delayed telling (ss)	90.39 (24.95)	101.71 (25.66)	82.82 (11.88)	107.33 (17.68)		3.14	0.04	0.18	ASD<T
	Comprehension (ss)	92.06 (20.98)	79.14 (36.97)	86.64 (22.61)	106.42 (15.74)		2.47	0.08	0.14	No differences
	MLUw (ss)	93.39 (16.33)	102.71 (22.57)	98 (26.88)	93.5 (18.59)		.44	0.23	0.03	No differences
Phonological awareness	Spoonerisms (*n* correctly transformed)	10.56 (9.57)	19.29 (6.94)	17.36 (10.01)	22.5 (1.73)		5.75	0.002	0.28	SLI<T
Verbal short-term-memory	Non-word repetition (rs)	24.55 (11.33)	36.42 (3.82)	33.90 (8.79)	41.08 (3.42)		9.93	<0.001	0.4	SLI<(PLI = ASD = T)
	Sentence repetition (rs)	19.11 (5.67)	23.71 (3.59)	22.18 (4.73)	29.16 (2.52)		11.83	<0.001	0.45	(SLI = ASD)<T
Pragmatic language a	Structural language composite (SLC) (rs)	24 (12.96)	12.57 (8.77)	25.36 (13.94)	0.6 (1.07)		11.8	<0.001	0.48	T<(ASD = SLI)
	Pragmatic language composite (PLC) (rs)	19.29 (9.28)	21.29 (11.21)	37.27 (10.93)	0.5 (0.85)		28.66	<0.001	0.69	T<(SLI = PLI)<ASD
	Structural–pragmatic mismatch (rs)	4.36 (6.83)	−7.71 (8.92)	−11.91 (10.66)	0 (0.92)		11.5	<0.001	0.44	(ASD = PLI)<SLI

Notes: rs, Raw scores; ss, standard scores.

a SLI = 14; PLI = 7, ASD = 11, Typical = 10. A high score denotes impairment.

### NVIQ

A one-way ANOVA revealed a main effect for group. The mean NVIQ of the three clinical groups were well within the normal range. However, in the case of the SLI group, this was significantly below that of the typical group.

### Structural language

Performance differences on the Goldman–Fristoe, BPVS-II and TROG-E were tested with multivariate analysis of variance (MANOVA) and Bonferroni *post-hoc* tests. There was an overall effect for group, *F*(9, 102) = 5.62, *p*<0.001, Wilks' *λ* = 0.38, partial eta^2^ = 0.28, as well as individual between-group differences for each task. Relative to the typical group, the SLI group demonstrated more articulation errors (Goldman–Fristoe), less grammatical knowledge (TROG-E) and reduced vocabulary (BPVS-II). The ASD group also had lower levels of vocabulary compared to the typical group. No further differences were found.

The four measures obtained from the ERRNI were then compared between groups. MANOVA revealed a significant effect of group, *F*(12, 109) = 2.11, *p*<0.05, Wilks' *λ* = 0.57, partial eta^2^ = 0.17. However, despite the group difference, effect sizes for the between-groups comparison on each task were small (for each comparison, partial eta^2^<0.2). Bonferroni *post-hoc* tests revealed one difference only: for the measure of delayed telling, the ASD group did significantly worse than the typical group.

### Phonological awareness and verbal STM tasks

MANOVA revealed a significant main effect of group, *F*(9, 102) = 4.1, *p*<0.001, Wilks' *λ* = 0.47, partial eta^2^ = 0.22. The SLI group had significantly lower scores than the typical group on all three tasks. The ASD participants performed poorly on the memory for sentences task, but had significantly higher scores than the SLI group on the task of non-word repetition.

### Pragmatic ability

CC-A data were missing for four participants in the SLI group and two participants in the control group (all of whom did not return the questionnaire). Good reliability was found for the subscales relating to the SLC and those contributing to the PLC (for both, Cronbach's *α*>0.9). These two measures were then compared between groups using MANOVA and Bonferroni *post-hoc* tests. There was an overall main effect for group, *F*(6, 74) = 15.98, *p*<0.01, Wilks' *λ* = 0.19, partial eta^2^ = 0.56, and an individual group effect for each measure (*p*s<0.001). Very few participants in the typical group obtained other than optimal scores on any of the checklist items and both the SLC and PLC of this group approached zero. The ASD and SLI groups (but not the PLI group) rated significantly higher than the typical group on the SLC, while all three clinical groups had significantly higher scores on the PLC. The ASD group rated particularly highly on the PLC, with a score that was significantly greater than both the SLI and PLI groups.

**Figure 1:**
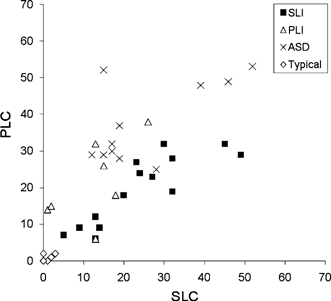
Scatterplot showing the relationship between the structural language composite(SLC) and the pragmatic language composite (PLC) of the Communication Checklist — Adult (CC-A). Higher scores on each index indicate greater deficit.

Univariate ANOVA found the structural–pragmatic mismatch of both the PLI and ASD groups significantly lower than that of the SLI group, indicating a disproportionate level of difficulty with social communication. As can be seen in Figure 1 where the SLC is plotted against the PLC, most children in the ASD and PLI groups had greater levels of pragmatic difficulties relative to structural language difficulties. There was a tendency for the ASD participants to have greater levels of deficit. In comparison, participants in the SLI group tended to have similar levels of structural and pragmatic language difficulties.

### Literacy ability

The performance of the four groups on the literacy tasks were analysed with MANOVA and Bonferroni *post-hoc* tests (Table 3). There was a main effect for group, *F*(21, 110) = 3.29, *p*<0.01, Wilks' *λ* = 0.25, partial eta^2^ = 0.37, as well as between-group differences for each task. The SLI group had significantly worse performance than the typical group on all indices of literacy ability. In comparison, the only tasks in which the ASD group performed significantly worse than the typical group were the real-word subtest of the TOWRE and the error total on the nonsense passage reading task. The PLI group demonstrated even better performance, scoring significantly below the typical group on the real word subtest of the TOWRE only.

**Table 3:** Mean performance (standard deviation) of the specific language impairment (SLI), pragmatic language impairment (PLI), autism spectrum disorder (ASD), and typical (T) groups on the battery of literacy tests. Only statistically significant differences are presented

Assessments	SLI (*n* = 18)	PLI (*n* = 7)	ASD (*n* = 11)	T (*n* = 12)	*F*	*p*	Partial eta^2^	Statistical significant differences
OSCCI spelling test (*n* words spelt correctly)	22.94 (9.66)	30 (9.68)	27.45 (7.23)	36 (3.38)	6.58	<0.01	0.31	SLI<T
TOWRE real word (ss)	72.22 (5.8)	79.14 (7.9)	83.36 (14.36)	94.75 (10)	13.32	<0.01	0.48	(SLI, PLI, ASD)<T; SLI<ASD
TOWRE non-word (ss)	68.72 (14.61)	86.39 (7.13)	91.18 (18.76)	98.17 (14.44)	10.85	<0.01	0.43	SLI<(T = PLI = ASD)
Proof reading (*n* errors correctly identified)	5.44 (3.09)	8.43 (4.28)	7.73 (3.1)	9.58 (1.88)	4.85	<0.01	0.25	SLI<T
Proof reading (time, seconds)	111.46 (33.1)	77.21 (27.12)	83.46 (15.68)	66.68 (21.28)	7.87	<0.01	0.35	SLI<(T = PLI = ASD)
Non-word passage (*n* errors)	21.94 (10.15)	13.29 (11.34)	20.5 (21.95)	6.67 (3.75)	3.8	<0.05	0.21	(SLI = ASD)<T
Non-word passage (time, seconds)	126.89 (49)	77.99 (37.07)	69.64 (41.63)	59.07 (13.59)	8.9	<0.01	0.38	SLI<(T = PLI = ASD)

### Discriminant function analysis

We then conducted a discriminant function analysis to establish whether the three clinical groups, who were categorized on childhood characteristics, could be distinguished from each other based upon language abilities at adulthood. The most salient measures of structural language (TROG-E, BPVS-II), verbal STM (memory for sentences), pragmatic language (PLC), reading (TOWRE phonemic decoding) and spelling (OSCCI spelling test) were entered into a discriminant function analysis as independent variables in stepwise fashion. Because of missing CC-A data, five participants in the SLI group and two participants in the typical group were excluded from this analysis. A significant discriminant function was found, Wilks' *λ* = 0.09, *χ*^2^(12, *n* = 42) = 90.34, *p*<0.001. All of the participants in the typical group were classified together. The majority of SLI cases were also classified together (69.2%). While the majority of PLI and ASD cases fell into distinct categories, there was some overlap between the groups; 71.3% of the PLI participants were classified together in one group (along with 36.4% of the ASD group) and 63.6% of the ASD were grouped in another (along with 28.7% of the PLI group). One SLI participant was categorized in each of these groups, while three SLI participants were classified with the group of typical participants. The largest contribution to the function was provided by the PLC.

### Change over time: linguistic and cognitive functioning

#### Non-verbal IQ

Paired *t*-tests were conducted separately for each group to examine change in mean non-verbal IQ over time. There was little change in the mean NVIQ of the SLI group (T1: mean = 101.28, SD = 10.9; T2: mean = 97, SD = 16.96, *t*(17) = 1.09, *p* = 0.29), the PLI group (T1: mean = 108.86, SD = 13.66; T2: mean = 105.71, SD = 16.58, *t*(6) = 0.53, *p* = 0.61) and the ASD group (T1: mean = 101.23, SD = 17.18; T2: mean = 103.36, SD = 19.35, *t*(11) = 0.52, *p* = 0.61). In comparison, the mean NVIQ of the control participants significantly increased from T1 (mean = 103.42, SD = 8.71) to T2 (mean = 118.5, SD = 8.61)*, t*(11) = 3.55, *p*<0.01.

#### Linguistic functioning

Table 4 shows the change in the linguistic severity scores from T1 to T2. The table confirms that the SLI participants had considerable structural language difficulties in childhood, with 14 of the 18 participants in this group deemed to have poor language functioning. Linguistic deficits persisted into adulthood for the majority of SLI participants, with the same number of cases presenting with poor linguistic functioning at T2. However, it is noteworthy that two participants who had poor linguistic ability in childhood, presented with good language functioning in adulthood. There was considerably more instability in the PLI group; three of the seven participants improved in linguistic ability and two participants had worse linguistic functioning at T2. Five of the eleven ASD participants had persisting language deficits, while a number of participants in this group improved from poor linguistic ability at T1 to fair (*n* = 3) or good linguistic functioning (*n* = 2) at T2. The only clinical participant (with ASD) who demonstrated good linguistic functioning in childhood presented with considerable linguistic deficit in adulthood.

**Table 4:** Change in linguistic ability over time

		T2		
		
		Poor	Fair	Good
T1	Poor	11 SLI; 5 ASD, 1 PLI	1 SLI, 1 PLI; 4 ASD	2 SLI, 1 PLI, 1 ASD
	Fair	3 SLI, 2 PLI	1 PLI, 1 Typical	1 SLI, 1 PLI, 1 Typical
	Good	1 ASD	2 Typical	8 Typical

Note: ASD, autism spectrum disorder; PLI, pragmatic language impairment; SLI, specific language impairment.

## Discussion

The findings indicate that the language profile of different subtypes of developmental language disorder — namely, pragmatic language impairment (PLI) and specific language impairment (SLI) — relates to language and literacy outcome in adulthood. Individuals with a childhood history of SLI were found to have lasting difficulties with speech production, receptive grammar, verbal short-term memory, and phonological awareness as well as considerable literacy impairment. In contrast, the participants in the PLI group showed relatively intact structural language and literacy ability, but had enduring difficulties with pragmatic language. Discriminant function analysis found that the participants in the SLI and PLI groups could be distinguished from each other based upon their performance on the language and literacy tasks administered at follow-up.

There were many similarities in the language profiles of the PLI and ASD participants, with both groups performing within normal limits on many of the structural language tasks. The one measure that did differentiate these groups was the pragmatic scale of the Communication Checklist — Adult (CC-A); the PLI group showed moderate difficulties, while the ASD had severe impairment. Whitehouse *et al.* (submitted), who explored the psychosocial outcomes of this participant sample, present a similar picture; the PLI group had some difficulty establishing social relationships and with stereotyped behaviours, but these were considerably more severe in the ASD group. These findings support the view that PLI and ASD are related disorders, but differ subtly along qualitative dimensions of language functioning and social interests (Bishop 2000).

Intriguingly, while there were clear differences between the four groups on many of the standardized assessments, the measures from the Expression, Reception and Recall of Narrative Instrument (ERRNI) did not appear to have the same discriminating power. There was no group difference on the measures of initial telling, narrative comprehension and mean length of utterance (MLU), and only a marginal difference between the ASD and the Typical group for the delayed telling measure. While the ERRNI provided information concerning the general narrative ability of the four groups, the current findings indicate that this measure may not be a sensitive tool for differentiating between different communication disorders.

Parent-report data obtained from the CC-A provided additional insights into the communicative ability of the three clinical groups. First, the SLI and PLI groups were reported to display equivalent levels of pragmatic difficulties. However, whereas the PLI group tended to show pragmatic impairment in the presence of relatively intact structural language, there appeared to be a linear relationship between the SLC and PLC in the SLI group, such that the level of pragmatic difficulties increased commensurate with the level of structural language deficit (Figure 1). The finding replicates that of Mawhood *et al.* (2000), who found that children with SLI had significant pragmatic difficulties when reassessed in early adulthood. However, the current finding has added importance in that the majority of children in the SLI group received an assessment in childhood that excluded for the presence of significant pragmatic impairment. What could explain the discrepancy between childhood and adulthood? One possibility is that pragmatic difficulties were in fact present during childhood but were subtle in nature and only become apparent when these individuals were exposed to increasingly demanding and complex social situations in adolescence and adulthood. An alternative possibility is that the pragmatic difficulties of the SLI participants may have arisen as a secondary consequence of the primary impairment in structural language. Language plays a critical role in social interaction and any impairment in the ability to communicate is likely to place a child at increased risk of social failure (Brinton and Fujiki 1993). Language difficulties may lead a child to withdraw from social situations, thus limiting their opportunities to participate in important social learning experiences. There is emerging evidence for this proposal, with Durkin and Conti-Ramsden (2007) finding strong associations between childhood language difficulties and social behaviour problems in adolescence. However, whether the pragmatic difficulties arose in response to the increasingly complex social environment or as a consequence of accumulated social failure, the current data indicate a clear need for social skills training programmes for adolescence and adults with SLI.

Another intriguing finding was that, despite the ASD participants performing within normal limits on the tests of articulation and grammar comprehension, parent report on the CC-A indicated structural language problems in this group that were at least as severe as the SLI participants. While formal language assessments provide a measure of specific linguistic domains (for example, articulation or grammar comprehension), the CC-A assesses how individuals use these abilities in a conversational context. The discrepancy between the findings of the formal language tests and the CC-A suggests that adults with high functioning ASD have a relatively strong base of structural language knowledge, but may have difficulty using these abilities in a functional capacity. Importantly, we caution against generalizing these outcomes to low functioning individuals, many of whom present with substantial structural language and literacy difficulties in adulthood (Howlin *et al.*^2004^).

The inclusion of the ASD group also allowed for an examination of contemporary ideas of the possible aetiological overlap between SLI and autism. Children with SLI have been found to perform poorly on tasks of non-word repetition, indicating limitations in phonological short-term memory (Gathercole and Baddeley 1990). Phonological short-term memory deficits are thought to be at the core of SLI and poor non-word repetition persists into adulthood for many individuals with SLI (Clegg *et al.*^2005^). Recent evidence that language-impaired children with autism also perform poorly on tasks of non-word repetition has led to suggestions that there may be some shared aetiological basis for the two disorders (Tager-Flusberg and Joseph 2003). An alternative view is that language impairments in autism are not caused by deficits in phonological short-term memory, and that the poor non-word repetition of these children is due to a more global cognitive impairment (Whitehouse *et al.*^2007^, ^2008^). If poor phonological short-term memory is at the core of the language impairment in autism (as is the case in SLI), then we would expect to observe non-word repetition deficits in adults as well as children with this disorder. This was not found in the current study; while the SLI group had considerable impairment on the non-word repetition task, there was no significant difference between the performance of the ASD and the typical group. Unfortunately, T2 cross-sectional only were available for this task, leaving open the possibility that the two groups data did not have equivalent non-word repetition performance at T1. Future studies can build on this work by comparing this ability between populations at several time-points across development.

While the longitudinal nature of the data is a strength of this study, the design also includes a number of limitations. Because the childhood studies were not originally intended to be prospective, participant numbers in the current study were dependent upon how many individuals could be traced successfully. The resulting group sizes were small, limiting the extent to which the results can be generalized. The variability in psychometric tests used between cohorts at T1 as well as between T1 and T2 also introduced the risk that measurement error may have influenced the findings. This was somewhat unavoidable due to the current lack of language tests that are standardized to adulthood. Importantly, however, the study also included a typically developing control group, made up of individuals from two different cohorts. Additionally, the large variability in chronological age at T1 (and T2) makes it difficult to determine the exact developmental trajectories of each group. Studies that systematically track a cohort of children from the same age group over time (for example, Manchester Language Study; Conti-Ramsden *et al.*^2001^) will contribute greatly to this goal. Finally, although we divided the language-impaired participants into distinct groups for analytic purposes, the mild structural language deficits of the PLI group and moderate pragmatic difficulties of the SLI group, indicate that there is no clear-cut diagnostic boundary between these language-impairment subtypes. The data presented here suggest that these labels may be best viewed as descriptors of broad areas of deficit rather than mutually exclusive diagnostic categories.

Only 48 of the original 130 participants were recruited and tested in this study, leaving open the possibility of recruitment bias. The contact details of a considerable proportion of T1 participants were destroyed after the original studies and consequently many of these individuals could not be located in adulthood (57 of 130). It is possible that the sample recruited at follow-up was skewed towards including a greater proportion of ‘linguistically able’ participants, either because these individuals felt more comfortable than others with their ability being scrutinized, or because they had a better understanding of what the study entailed. Although we guarded against this to the best of our ability (for example, including an information DVD to familiarize potential participants with the study demands), the retrospective recruitment procedure made sample bias somewhat inevitable. A similar kind of recruitment bias appears to have occurred with the Typical group, which had a mean non-verbal IQ and receptive vocabulary at T2 that was well above the population average. Note, however, that this has little impact upon the current findings; the low scores of the clinical groups (predominantly the SLI group) were often well below the population mean of the standardized assessments.

Despite these limitations, clear results emerged regarding the long-term outcome of children with different patterns of language impairment. Childhood deficits persisted over time, so that the majority of children with SLI presented with deficits in structural language and literacy in adulthood, while children with PLI had primary difficulties with pragmatic language. These findings highlight the importance of a wide-ranging clinical assessment at childhood that takes into account qualitative aspects of communication as well as language test scores. Gaining an understanding of communicative ability across the entire language profile will afford clinicians greater insight into the possible outcomes of different children, which will assist in the identification of areas likely to be in need of current or future intervention.

## Notes

1 There are a number of recognized subtypes of developmental language disorder, including phonological disorder, expressive language disorder, and mixed receptive/expressive language disorder. Note, however, that the current study is concerned with the broader discrimination between these ‘linguistic’ difficulties (structural language impairment) and social language problems (pragmatic language impairment).

2 The ADI-R is a semi-structured parent interview conducted by trained examiners (in the current study, H. F., D. B. and A. W.), which asks for information about their child's behavioural development, with a particular focus on the period between 4 and 5 years of age. Parents' responses are coded and then converted to numerical scores, which provide the basis for determining whether individuals exceed the designated cut-off for an autism diagnosis. All participants reclassified into the autism group met this threshold in each of the three ‘autistic’ domains (communication, socialization, repetitive behaviour). The ADI-R is not conventionally used as a retrospective diagnostic tool and there are limitations to this approach (for example, contamination of parent recall). For more information about this procedure, including its limitations, see Bishop *et al.* (2008).

3 Three participants included in Bishop *et al.* (2008) were excluded from the current report as they were under 16 years at follow-up.

## Appendix

**Appendix A:** Studies in which the current participants took part in childhood

	Cohort					
	
	A	B	C	D	E	F
Research	Analysis of conversational ability in language impairment	Analysis of conversational ability in PLI	Analysis of conversational ability in language impairment	Electrophysiological study of auditory processing in SLI	Behavioural investigation of borderlands of SLI and Autism	Behavioural investigation of borderlands of SLI and Autism
Reference	Adams and Bishop (1989)	Bishop *et al.* (1994)	Bishop *et al.* (2000)	McArthur and Bishop (2004)	Bishop and Norbury (2002)	Norbury *et al.* (2004)
Assessments at T1	*Non-verbal***Block Design and Picture Completion subtests of WISC-R**	*Non-verbal***RCPM**	*Non-verbal***RCPM**	*Non-verbal***RSPM**	*Non-verbal***RCPM**	*Non-verbal***WASI PIQ**
	*Linguistic***WISC-R-VC** TROG MLU	*Linguistic***BPVS** ITPA	*Linguistic* CELF-R-RS **BPVS** MLU	*Linguistic***BPVS-II** CELF-R-RS TLC-RS	*Linguistic***BPVS-II** TROG-2 CELF-R-RS	*Linguistic***BPVS –II** CELF-III-CD CELF-III-RS
	*Pragmatic* Teacher report	*Pragmatic* Teacher report Precursor to CCC	*Pragmatic* Teacher report Precursor to CCC	*Pragmatic* Not assessed	*Pragmatic* CCC	*Pragmatic* Teacher and/or clinician report CCC-2
Specific language impairment (SLI) diagnosis	Based upon teacher and researcher judgement	Not recruited	Scored 1 SD below the mean on at least one linguistic testScoring ≤10 on CCC precursor	Scoring more than 1 SD below the age-expected level on at least two linguistic tests	Scored 1 SD below the mean on at least one linguistic testScoring > 132 on pragmatic composite of CCC	Standard scores more than −1.25 SD below the mean on two of the three linguistic tests
Pragmatic language impairment (PLI) diagnosis	Based upon teacher and/or researcher judgement	Initially identified by teachers, researchers and/or clinicians Scoring ≥12 on CCC precursor	Initially identified by teachers, researchers and/or clinicians Scoring ≥12 on CCC precursor	Not recruited	Scoring<133 on pragmatic composite of CCC	Teacher and/or clinician judgement of significant pragmatic difficulties Confirmed via CCC-2

Notes: All participants had a non-verbal IQ within the normal range. The measures used as an index of ability at T1 are shown in bold.

BPVS, British Picture Vocabulary Scale (Dunn *et al.*^1982^); BPVS-II, British Picture Vocabulary Scale — Second Edition (Dunn *et al.*^1997^); CCC, Children's Communication Checklist (Bishop 1998); CCC-2, Children's Communication Checklist — Second Edition (Bishop 2003); CELF-R-RS, Recalling Sentences Subtest from the Clinical Evaluation of Language Fundamentals — Revised (Semel *et al.*^1987^); CELF-III-CD, Concepts and Directions Subtest from the Clinical Evaluation of Language Fundamentals — Third Edition (Semel *et al.*^1995^); CELF-III-RS, Recalling Sentences Subtest from the Clinical Evaluation of Language Fundamentals — Third Edition (Semel *et al.* 1995); ITPA, Illinois Test of Psycholinguistic Ability — Revised Edition (Kirk *et al.*^1968^); MLU, mean length of utterance; PLI, pragmatic language impairment; RCPM, Raven's Coloured Progressive Matrices (Raven *et al.*^1986^); RSPM, Raven's Standard Progressive Matrices (Raven *et al.*^1992^); SLI, specific language impairment; TLC-RS, Recreating Sentences Subtest from the Test of Language Competence — Expanded Edition (Wiig and Secord 1989); TROG, Test for Reception of Grammar (Bishop 1989); WASI PIQ, Wechsler Abbreviated Scales of Intelligence — Performance IQ (Wechsler and Chen 1999); WISC-R, Wechsler Intelligence Scales for Children — Revised (Wechsler 1974); and WISC-R-VC, Verbal Comprehension Subtest of the Wechsler Intelligence Scales for Children — Revised (Wechsler 1974).

**Appendix B:** Structural language severity ratings at initial testing (T1) and at follow-up (T2)

Severity ratings
T1: Cohorts A, C, D, E, F = good, within normal limits on the three linguistic tasks administered; fair, within normal limits on two tasks; and poor, within normal limits on one or no tasks. Cohort B = good, within normal limits on the two tasks administered; fair, within normal limits on one; and poor, within limits on no task.
T2: Good: within normal limits on the BPVS-II (raw score ≥134), TROG-E, MLUw and memory for sentences task from the NEPSY; fair, within normal limits on three of these tasks; poor, within normal limits on two or less of these tasks.
